# Prelysosomal Compartments in the Unconventional Secretion of Amyloidogenic Seeds

**DOI:** 10.3390/ijms18010227

**Published:** 2017-01-23

**Authors:** Helena Borland, Frederik Vilhardt

**Affiliations:** 1 Department of Neurodegeneration In Vitro, H. Lundbeck A/S, 2500 Valby, Denmark; HBOM@lundbeck.com; 2 Department of Cellular and Molecular Medicine, Panum Institute, University of Copenhagen, 2200N Copenhagen, Denmark

**Keywords:** autophagosomes, α-synuclein, late endosomes, unconventional secretion, neurodegeneration

## Abstract

A mechanistic link between neuron-to-neuron transmission of secreted amyloid and propagation of protein malconformation cytopathology and disease has recently been uncovered in animal models. An enormous interest in the unconventional secretion of amyloids from neurons has followed. Amphisomes and late endosomes are the penultimate maturation products of the autophagosomal and endosomal pathways, respectively, and normally fuse with lysosomes for degradation. However, under conditions of perturbed membrane trafficking and/or lysosomal deficiency, prelysosomal compartments may instead fuse with the plasma membrane to release any contained amyloid. After a brief introduction to the endosomal and autophagosomal pathways, we discuss the evidence for autophagosomal secretion (exophagy) of amyloids, with a comparative emphasis on Aβ_1–42_ and α-synuclein, as luminal and cytosolic amyloids, respectively. The ESCRT-mediated import of cytosolic amyloid into late endosomal exosomes, a known vehicle of transmission of macromolecules between cells, is also reviewed. Finally, mechanisms of lysosomal dysfunction, deficiency, and exocytosis are exemplified in the context of genetically identified risk factors, mainly for Parkinson’s disease. Exocytosis of prelysosomal or lysosomal organelles is a last resort for clearance of cytotoxic material and alleviates cytopathy. However, they also represent a vehicle for the concentration, posttranslational modification, and secretion of amyloid seeds.

## 1. Introduction

The gradual decline of neuronal populations in protein malconformation brain disease is due to the direct or indirect neurocytopathic effects of misfolded and aggregate-prone endogenous nerve cell proteins. These disease-specific proteins include the prion protein (PrP^sc^) of Creutzfeldt-Jakob disease, α-synuclein, which is the most abundant protein in Lewy Body inclusions of Parkinson’s disease (PD), superoxide dismutase (SOD) in Amyotrophic Lateral Sclerosis, and in Alzheimer’s disease (AD) hyper-phosphorylated tau makes up neurofibrillary tangles, while cleavage of amyloid precursor protein (APP) causes parenchymal deposition of amyloid β peptide (Aβ) in the form of amyloid plaques [1]. Despite lack of sequence homology, these proteins form aggregates in the form of oligomers and fibrils, which present a similar pathological folding epitope consisting of a “steric zipper” of β-pleated sheets [2], and they are in a sense self-replicating if enough monomer protein is present to sustain misfolding and the aggregation cascade [1]. For the extent of this review, we will use the term amyloid to refer to multimeric proteinaceous assemblies with distinctive histochemical features in tissues, and a cross-β quaternary structure as determined by X-ray fibre diffraction analysis [1]. Different species of oligomers and fibrils of any specific amyloid can exert neurotoxicity by different mechanisms, and multiple inoculating species of each amyloid are capable of propagating the disease [3,4]. Nerve cells will suffer from intracellular protein aggregation, as well as pathological interference by secreted species of amyloid mainly in soluble oligomer form [5,6,7], but also fibrils [8]. Typical neurocytopathic features of intracellular protein aggregation includes inhibition of degradative systems (autophagolysosomal pathway and the proteasome), development of Endoplasmatic reticulum (ER)-stress, and induction of mitochondrial and cytosolic oxidative stress, which, if sustained, result in nerve cell death.

In the following we will first briefly discuss seeded conversion and transmission of amyloid proteopathy, and the basic working of the endosomal and autophagosomal pathways, before we finally delve into the trafficking of prelysosomal organelles and their possible role in secretion of amyloid and propagation of amyloid proteopathy in the brain.

## 2. Seeded Conversion and Transmission of Amyloid Proteopathy In Vivo and In Vitro

### 2.1. Seeded Conversion

Amyloid formation has almost universally been shown to involve a nucleation-dependent polymerization reaction [1,9], which also describes well-characterized biological processes such as microtubule assembly and actin polymerization. First, a so-called nucleus forms from a few assembled monomers in a misfolded state. This initial step is highly thermodynamically unfavorable and is rate limiting for amyloid growth. Once a nucleus has emerged, it catalyzes, in a prion-like fashion, the seeded conversion of endogenous, monomeric protein into a pathological conformational state, which is competent for polymerization into oligomers and then fibrils provided sufficient monomeric, wild type protein is present [1,4,9,10,11]. As such, seeded conversion is the driving force of template-dependent propagation of amyloids in the brain. Total amyloid growth is accelerated by secondary nucleation events caused by fibril breakage, which generates more free ends for templating and polymer growth [10,11,12]. Each of the three microscopic steps of aggregate formation, namely nucleation, fibril growth, and fibril amplification, is to a different degree influenced by environmental factors such as lipid bilayers [11] and pH [12], as well as by disease-causing mutations [11]. The kinetic reactions are very complex to solve analytically and only recently has it been possible to design a toolkit for the mathematical description of filament nucleation and growth. The results underpin the long lag phase for aggregate formation, reflecting the rare event of nucleation, and the accelerated amyloid growth obtained by fragmentation and secondary nucleation [10]. Although a description of the seeding process is approaching, the underlying mechanism(s) of amyloid formation is still being debated.

Each given amyloid may adopt distinct conformational states resulting in fibrils with different structural, chemical, and pathological properties [13,14,15,16]. The molecular composition and conformation of specific amyloid strains can be maintained by seeded conversion both in vitro and in vivo [17]. For each patient one strain dominates the pathology, dictated by the rarity of the nucleation event [14], which likely contributes to the diverse clinical manifestations of AD and PD in patients. In mouse models one species of amyloid can influence aggregation of other amyloids, a phenomenon referred to as cross-seeding, and this precipitates a particularly rapidly progressing neurodegeneration in vivo [18,19]. The results are relevant to the development of PD-associated dementia, part of which, is most likely caused by incipient AD [20].

### 2.2. The Unit Operations: Release, Transmission, and Uptake

Within the past few years, an additional layer of knowledge has been added to our understanding of protein malconformation brain disease. Evidence gained from research in cultured nerve cells and from experimental mouse models of AD, PD, and tauopathy indicates that these neurodegenerative diseases spread in the brain by the transcellular relay (transmission) of amyloid protein in possibly multiple inoculating forms [21], from neuron to neuron [1,4,22,23,24]. Secreted amyloid from an affected neuron can gain entry into the cytosol of a neighbouring neuron (or glia cell for that matter), and function as a proteopathic template for recruitment of endogenous protein monomers into the pathological cascade of protein misfolding and aggregation.

Our knowledge about the cellular and molecular mechanisms that mediate critical unit operations of the proposed transmission model of proteopathy is clearly lagging behind the strong phenomenalistic evidence from animal models. Unit operations must, as a minimum, include secretion of amyloid from donor cell to extracellular milieu, intercellular transfer, and finally, entry into recipient cell cytosol to initiate templated protein misfolding and perpetuation of disease.

The release of Aβ_1–42_ from nerve cells following proteolytic cleavage of the APP membrane protein, and the secretion of PrP^sc^ from infected cells, have been recognized for long, but more recently secretion of cytosolic amyloid species from cultured nerve cells has been documented for tau [25,26], α-synuclein [27,28,29,30,31,32,33] and SOD [34,35].

A growing number of in vitro studies have demonstrated the potential of monomeric, oligomeric, and/or fibrillary forms of amyloid to be taken up by neurons in culture, including α-synuclein [36,37,38,39,40], tau [41,42], Aβ_1–42_, and SOD [43]. While monomeric species of amyloid may pass membranes passively [39], there is evidence of active endocytic uptake mechanisms for aggregated tau [41,42] and α-synuclein species in nerve cells [37,38,39]. At least α-synuclein uptake can be blocked by dynamin inhibition [37,38,39] suggesting receptor-mediated uptake trough pinocytic routes, although, at least one study indicated a dynamin-independent entry route [44]. Aggregated SOD is potentially internalized via macropinocytosis [43]. Heparan sulphate proteoglycans on the surface of cells may bind amyloids for internalization, or more likely, transfer amyloid to an internalization receptor for entry akin to virus internalization [45]. However, no internalization receptors for amyloid have been defined in neurons, although surface receptors are known [46]. In contrast, microglia, the supportive glia cells and resident macrophages of the brain [47], express a number of immune cell receptors, which have been implicated in the uptake of amyloids into microglia and/or inflammatory activation of microglia. Some of these receptors e.g., Fc-γ receptors for IgG (FcγR) and Toll-like receptors (TLRs) are also expressed at lower levels in neurons. In microglia α-synuclein is internalized by TLR4 [48,49] and perhaps FcγR [50], while activation (cytokine production) of microglia depends on TLR2 [51,52] and potentially FcγR’s [50,53]. In addition, morphological changes and chemotactic response of microglia towards aggregated α-synuclein is mediated through CD11b (integrin α_2_) [54] and β1 integrins [55]. In the context of AD many different receptors have been implicated in immune cell activation and internalization of Aβ_1–42_ by microglia including scavenger receptor, receptor for advanced glycation endproducts (RAGE), CD36 in a complex with CD47 and α_6_β_1_ integrin, CD14, CD11b/CD18 (α_2_β_m_), and TLR2 and 4 [56,57,58,59,60,61,62,63,64,65,66,67]. Uptake might be either pinocytic or macropinocytic [68], and uptake of exosome-associated Aβ_1–42_ has been reported [69].

In vitro and in vivo experiments demonstrate interneuronal transmission of endogenously produced and secreted α-synuclein [27,36,37,40,70,71,72], tau [25,41,73,74,75,76], SOD [35,43], or Aβ_1–42_ [3,21,77,78,79,80]. In a number of the in vitro studies transmitted amyloid was found to seed aggregation and cytosolic inclusions of endogenous wild type protein in the acceptor nerve cells, and cytopathy could be propagated for extended passages [25,36,40,41,43,81]. A few reports on transmission of α-synucleinopathy with Lewy Body disease from host cells to engrafted fetal neurons in human PD have been published [70,82,83].

### 2.3. In Vivo Transmission of Proteopathic Amyloids

A series of simple in vivo experiments have provided solid evidence for the transmission of proteopathic amyloids in the brains of wild type or transgenic mice. Early experiments described the ability of brain homogenates containing pathological Aβ aggregates to inoculate cerebral amyloidosis following injection into the brains of APP transgenic mice [3,21,78,84]. Recently, propagating AD- or PD-like pathology and neurodegeneration in mouse brain were achieved by injection of purely in vitro preformed, recombinant Aβ_1–42_ or α-synuclein aggregates, respectively [71,72,80]. In a similar venue focal expression of mutant tau in mouse entorhinal cortex caused propagation of tau pathology to other anatomically interconnected brain areas along nerve fiber tracts [76], and injection of tau aggregates into the brain of nontransgenic mice is sufficient to precipitate propagating tau inclusion disease [85]. These experimental findings are in good agreement with the longstanding observation that spread of AD and PD pathology in the human brain follows a mainly stereotypical pattern along fiber tracts as delineated by Braak and colleagues [1,20,22,86]. Most recently, detailed analysis of the temporal progression of aggregation disease through synaptically connected neurons in wild type mice receiving α-synuclein fibril injections into the olfactory bulb has been presented in a new model of synucleinopathy, which may mimic aspects of prodromal PD [87]. Injection of brain extracts containing Aβ_1–42_ into the peritoneal cavity of mice inoculates vascular β-amyloidosis in the brain [78], and recombinant α-synuclein injected into peritoneum or blood stream finds its way to the brain parenchyme [8] demonstrating the potential of amyloids, other than PrP^sc^, for retrograde trafficking to the brain from the periphery. In the case of PD there is evidence that environmental toxin-induced α-synuclein misfolding can start in the enteric nervous system followed by retrograde transport of amyloids via the vagus nerve to the central nervous system [88].

Of the different amyloids only PrP^sc^ is considered a truly infectious agent as it can be passed between individuals (cannibalism) and even species (transmission of mad cow disease) after oral ingestion to inoculate disease in the recipient. It should be noted however, that retrospective studies of patient cohorts that received cadaveric pituitary growth hormone and became infected with Creutzfeldt-Jakob disease, also demonstrate a high proportion of AD pathology in the brain, indicating that both diseases were transmitted to the recipient [89].

After a short introduction of the endosomal and autophagosomal pathways, we will discuss the potential role of late endosome and amphisome exocytosis in the dissemination of proteopathic species of amyloid, with an emphasis on α-synucleinopathy.

## 3. The Endosomal Pathway

Early endosomes are the primary recipient organelle of endocytosed membrane lipid and protein. From early endosomes, which morphologically appear as tubulovesicular elements, the majority of internalized membrane and receptors are recycled to the plasma membrane within 20 min by direct and indirect sorting pathways [90]. During this time the bulk of the early endosome receives defining molecules by vesicular trafficking or binding of cytosolic factors to the endosome membrane that govern the gradual molecular maturation into a late endosome. Such molecules include Rab7, homotypic fusion and protein sorting (HOPS) complex, phosphatidylinositol 3-phosphate kinase (PI3K), endosomal sorting complexes required for transport (ESCRT)-0 and -I, Vacuolar Adenosine Triphosphatase (v-ATPase), and the mannose-6-phosphate receptor (M6PR). In addition, the endosome matures morphologically: membrane proteins, such as the epidermal growth receptor destined for degradation in lysosomes, are removed from the recycling pathway by a process of inward budding of the limiting endosomal membrane. This process forms multivesicular bodies (MVBs; a morphological term including late endosomes) containing intraluminal vesicles (ILVs) enriched by a receptor-mediated process for certain lipids and proteins [91,92]. The cargo is mainly destined for degradation in lysosomes, but a minor proportion will be released from cells by late endosome exocytosis. After secretion, ILVs are by convention termed exosomes, which, by their receptor-mediated inclusion of cytosolic cargo, can carry biological information (RNA) or even function (protein) between cells [93,94,95].

### 3.1. ESCRT-Dependent and -Independent Loading of Intraluminal Vesicles (~Exosomes)

Import of many protein cargoes into ILVs is dependent on mono- or poly-(with a preference for K63-linked) ubiquitinylation and is mediated by four cytosolic multi-component protein assemblies localized on the surface of MVBs called endosomal sorting complexes required for transport (ESCRT0-III) [92,96]. ESCRT-0 subunits hepatocyte growth factor-regulated substrate (Hrs)/signal-transducing adaptor molecule (STAM) are required for cargo selection and sorting. The functions of ESCRT-I and -II are more uncertain despite the presence of ubiquitin-binding domains [97] and may reside in the recruitment of ESCRT-III. This last complex is required for ILV abscission from the MVB membrane through recruitment of the AAA+ ATPase Vps4. But first, or concomittantly, ESCRT-III and Vps4 mediate de-ubiquitination of cargo and dissolution of the ESCRT complexes for new rounds of sorting [92,96]. Hrs of ESCRT-0 [98] and Vps proteins of ESCRT-III participate in different kinds of, potentially cargo-organized, protein lattices on the cytosolic side of the limiting membrane, which perhaps function in the receptor-mediated sequestration of lipids and proteins to be sorted [96]. Syndecan heparan sulphate proteoglycans and their cytosolic adaptor syntenin transfer cargo to the ESCRT machinery for sorting into ILVs in an Adenosine diphosphate-ribosylation factor 6 (ARF6) and phospholipase D-regulated mechanism [27,99,100,101,102]. Other examples of accessory proteins that can feed ubiquitinated substrate into the ESCRT pathway are known including the yeast orthologue of Alix [103].

Notably, other forms of sorting platforms formed by either ceramide or tetraspanins also function in the sorting to and formation of ILVs [91,104,105]. These ESCRT-independent sorting platforms seem to recruit cargo solely on the basis of biophysical properties such as aggregation, membrane association, and recruitment to cholesterol- and sphingolipid-enriched membrane microdomains [106]. The ESCRT-dependent and -independent mechanisms of ILV formation give rise to ILV populations of different sizes [107].

### 3.2. Final Endosome Maturation

The subtractive and additive trafficking of lipids and membrane proteins results in the gradual molecular and morphological maturation of the early endosome into a mature late endosome, which finally fuses with lysosomes for degradation of its luminal contents including ILVs. The fusion process is regulated by HOPS, which promotes tethering and fusion of membranes through interaction with the soluble *N*-ethylmaleimide-sensitive factor-attachment protein receptors (SNARE) fusion machinery. Specific SNAREs, including Vesicle transport through interaction with t-SNAREs homolog 1B (Vti1b), and syntaxins 7 and 8 on late endosomes, engage with the lysosomal SNARE vesicle-associated membrane protein (VAMP)8 to make up the *trans*-complex that mediate fusion of endosomes with lysosomes. The same Q-SNAREs can engage with VAMP7 to mediate preferentially homotypic fusion of late endosomes [108].

However, a minor proportion of MVBs/late endosomes may instead fuse with the plasma membrane under physiological conditions to release the luminal contents to the surroundings (see below). It is not known whether these exocytic MVBs constitute a specified subpopulation, but different forms of perturbation of the endocytic pathway or lysosomal deficiency increases secretion of exosomes [27,101,102].

## 4. The Autophagosomal Pathway

Autophagy is a lysosomal degradation pathway, which is responsible for the removal and degradation of cytosolic cargo including protein aggregates, long-lived proteins, and worn-out organelles and macromolecules [109,110,111,112]. Basal autophagy is cytoprotective and essential for neuronal function and survival because of the post-mitotic status of neurons (accumulated material cannot be diluted by cell division) [113,114]. However, autophagic flux can be increased further in response to either intracellular stress in the form of nutrient depletion, reactive oxygen species, mitochondrial dysfunction, ER-stress, and accumulation of certain cytosolic protein aggregates [109], or different kinds of external cues including pathogen invasion and inflammation [115].

In the following we will discuss macroautophagy (henceforth referred to autophagy), the autophagic mechanism quantitatively most important for the bulk disposal of macromolecular cargo. However, other forms of autophagy including micro-autophagy [116], and chaperone-mediated autophagy [117], which deliver autophagosomal substrates directly to the lysosome without vesicular autophagosome intermediates, are also relevant in the context of neurodegenerative disease.

### 4.1. Autophagosome Nucleation

An autophagosome is formed by the sequestration of a volume of cytosol by a double or multi-layered membrane [112,118]. The autophagosome is born as a phosphatidylinositol 3-phosphate (PI3P)-specified domain in conjunction with the ER called an omegasome because of its cradle-like structure [119,120]. Initiation is governed by the ser/thr-kinase uncoordinated family member-51-like kinase (ULK1/ULK2) initiator complex, which regulates a multiprotein complex containing Beclin-1 (Atg6), Atg14, and Vps34 (PI3K-III) with regulatory sub unit p150. PI3K activity is required for association of PI3P effectors, which in turn recruit essential autophagy machinery for nucleation of the omegasome [121]. The cellular growth regulator mammalian target of rapamycin (mTOR; inhibition of ULK) and the cellular energy charge sensor AMP-activated protein kinase (AMPK; activation of ULK) controls the ULK complex and autophagy initiation [109,110]. The omegasome develops by addition of membrane derived mainly from the ER lipid pool [119,120,121] into a so-called phagophore (also called isolation membrane). Also mitochondria, the Golgi apparatus, and the plasma membrane/early endosomes constitute reservoirs of lipid and membrane for the nascent autophagosomal organelle [122,123,124,125,126,127,128]. These organelles can be seen making contacts with the phagophore, sometimes multiple organelles at once [129]. Actin nucleation is required for formation and expansion of the omegasome and phagophore [130], and is likely also required at later stages of autophagy [131,132].

Elongation and expansion of the phagophore is regulated by a host of conserved *ATG* gene products [110] including the two protein conjugation systems Atg5-Atg12-Atg16L and Atg8. Conjugation of Atg5-Atg12 to autophagosomes is transient. In contrast, Atg8 homologues, consisting of two families defined by microtubule-associated protein 1 light chain 3 (LC3) and GABAA receptor-associated protein (GABARAP) are conjugated to phosphatidylethanolamine in both the inner and outer autophagosome membrane. LC3 and GABARAP remain associated with autophagosomes up to and including autolysosomes, making LC3 a commonly used marker of autophagosomes [112,118,133]. LC3 and GABARAP are required for phagophore expansion [133,134] where the membrane is delivered de novo, but also by the SNARE-regulated fusion of Atg9- or Atg16L-positive vesicles derived from the plasma membrane and the early endosome compartment [123,124,125,126,135]. Autophagosome precursors grow further in size by homotypic fusion in a SNARE-regulated process [136]. GABARAP acts at a later stage of autophagosome maturation than LC3 and influences autophagosome membrane closure [134], cargo sequestration [137], as well as final fusion with endolysosomes [138]. The six mammalian LC3/GABARAP homologues interact with a cohort of accessory proteins, some of which are carriers of autophagosomal cargo (see selective autophagy below), while others are regulators of the autophagosomal pathway [139].

Non-canonical forms of macroautophagy that work independently of one or more ATG gene products are known [127,140,141], as are mTOR-independent pathways of autophagy induction [142,143,144].

#### Selective Autophagy

Whereas starvation-induced autophagy indiscriminately engulfs, degrades, and recycles cytoplasmic constituents for anabolic reactions, selective autophagy, also called quality-control autophagy, accepts preferentially ubiquitinylated cargo. A number of autophagy receptors including p62/SQSTM1 [145] and SQSTM1-related proteins such as Neighbor of BRCA1 gene 1 (NBR1), optineurin, and Nuclear domain 10 protein 52 (NDP52) [146] bind poly-ubiquitinated cargo as well as LC3 and other Atg8 proteins thereby selectively recruiting ubiquitinated substrates to the phagophore for sequestration [147]. Autophagy receptors unrelated to p62 are also known [148]. These autophagy receptors are essential for the ability of cells to clear cytosolic protein aggregates. The proteasome also accepts ubiquitinylated cargo, and adaptor proteins like histone deacetylase 6 (HDAC6) [149] and Bcl-2 associated athanogene (BAG) proteins [150] are required for shuttling cargo to the autophagosome pathway during conditions of depressed proteasome activity or increased protein misfolding and ER-stress [111].

### 4.2. Autophagosome Maturation

The final destination of the contents enclosed by the autophagosome is the lysosome through the formation of an autolysosome. While this direct fusion event can occur, it has been recognized for some time that autophagosomes mature mainly by first fusing with endosomal compartments, including early (recycling), and in particular, late endosomes, before final fusion with lysosomes [151,152,153]. The hybrid compartment thus formed is termed an amphisome, which contains both cytosolic material taken up by autophagy as well as endocytic cargo including ILVs. It is therefore not surprising that many of the factors that govern autophagosome maturation and fusion with lysosomes derives from the endocytic pathway.

#### Autophagosome Mobility

Autophagosome transport depends on intact microtubules [154] and functional dynein-dynactin motors [155]. In nerve cells, most autophagosomes are formed distally in the neuritic network, which contains the bulk of the cytosol, and then transported retrogradely along microtubuli while maturing towards the cell soma where lysosomes are concentrated [156,157]. In this respect, the fusion of autophagosomes with late endosomes is functionally required for the amphisomal acquisition of dynein motor protein, and inhibition of fusion by syntaxin17 knock-down results in the axonal accumulation of autophagosomes [158]. By hooking up with different adaptors (Rab-interacting lysosomal protein (RILP) or FYVE and coiled-coil domain-containing protein 1 (FYCO)), Rab7 regulates plus- or minus-end directed motility of amphisomes and late endosomes on microtubule tracks through interactions with dynein-dynactin and kinesin motors, respectively [159,160,161,162,163,164]. Autophagosomes are decorated with both kinesin and dynein motors indicating that either motor can be silenced for vectorial transport [156]. HDAC6 binds polyubiquitin and dynactin, and is essential in the retrograde trafficking of ubiquitinated protein aggregates and organelles towards the cell center [165,166].

### 4.3. Autophagosome Fusion with Endolysosomes

**Figure 1:**
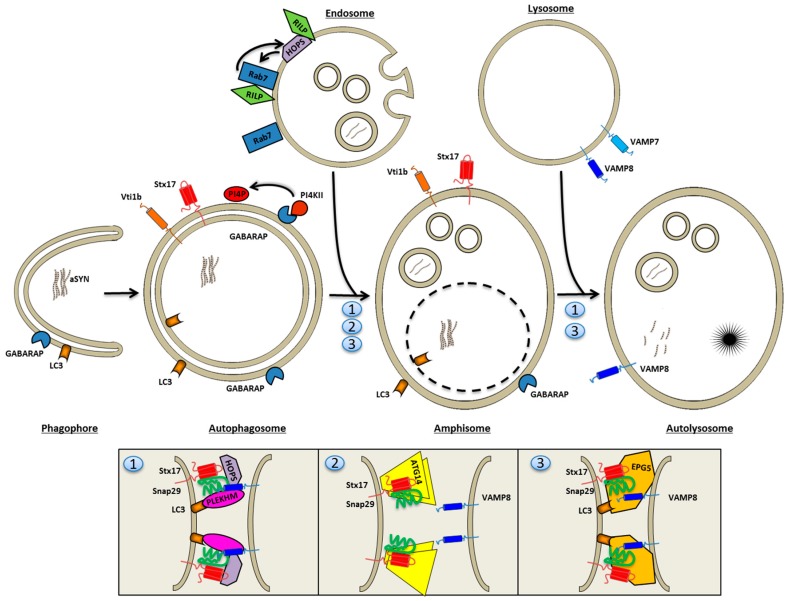
Entry of autophagosomes into the endolysosomal system. The closed autophagosome is contained by two membranes, which allows the ultrastructural distinction of autophagosomes from any other organelle, as the outer membrane is lost upon the first fusion reaction. The autophagosomal Soluble *N*-ethylmaleimide-sensitive factor-attachment protein receptor (SNARE) syntaxin17 and Atg14, which both influence later fusion reactions, are present on the phagosome Phosphatidylinositol 4-kinase (PI4K)II and its lipid product phosphatidylinositol 4-phosphate (PI4P) is specifically required for autophagososomes and late endosomes to form the hybrid organelle, an amphisome, in a fusion reaction mediated by Homotypic fusion and protein sorting (HOPS) and syntaxin17/Synaptosomal-associated protein (SNAP)29. Atg14 may primarily exert its function also at this stage. The subsequent fusion with lysosomes is also dependent on HOPS and syntaxin17/SNAP29, which partner up to form fusion competent trans-SNARE complexes with lysosomal SNARE Vesicle-associated membrane protein (VAMP)8. Alternative SNAREs for this fusion reaction are potentially v-SNARE Vesicle transport through interaction with t-SNAREs homolog 1B (Vti1b) and t-SNARE VAMP7. A number of factors have recently been identified that promote syntaxin17/SNAP29/VAMP8 interactions in different forms. ATG14 binds syntaxin17 directly, while Microtubule-associated proteins 1A/1B light chain 3B (LC3B) recruits both Pleckstrin homology domain-containing family M (PLEKHM) and Ectopic P-Granules Autophagy Protein 5 Homolog (EPG5) to the autophagosome. Here EPG5 (3) stabilizes the trans-SNARE complex with VAMP8, while PLEKHM (1) and ATG14 (2) interact with the bivalent SNARE pair syntaxin17/SNAP29.

Within the past few years, many groups have contributed novel insight into the identity of SNAREs required for fusion of autophagosomes with endosomal or lysosomal membranes, and the factors that regulate the fusion machinery (see Figure 1). Often the distinction between autophagosome fusion with late endosomes or lysosomes is not explicitly addressed experimentally, which is why many of these fusion factors are described as controlling entry into endolysosomes (meaning late endosomes and lysosomes). Nevertheless, the distinction is important. As we shall see, different conditions and different SNAREs are required for exocytosis of prelysosomal versus lysosomal elements, which becomes relevant in the context of disease-associated perturbations of the maturation pathways.

The fusion of autophagosomes with endosomes and the fusion of amphisomes with lysosomes is regulated by Rab7 [159,167,168,169], a central guanosine triphosphatase (GTPase) in the control of late endosome and lysosome mobility, distribution, and fusion [164,170,171]. An important Rab7 effector is the HOPS complex. Failure to recruit Rab7 to prelysosomal elements, pharmacologically obtainable by thapsigargin treatment, causes a block of autophagosome fusion with lysosomes [168].

The enzymatic activity of phosphatidylinositiol 4-phosphate kinase II (PI4KII) regulates the fusion between autophagosomes and late endosomes to form amphisomes [138]. GABARAP is required for recruitment of PI4KII, which generates phosphatidylinositol 4-phosphate (PI4P) on the surface of autophagosomes. Deficiency of the kinase or PI4P on the autophagosome specifically blocks fusion of autophagosomes with late endosomes as a double-layered membrane surrounds the organelles accumulating under these conditions [138]. The role played by PI4P for entry into the degradative endolysosomal compartment is currently unknown.

Syntaxin-17 is a SNARE that works exclusively in the autophagosomal pathway, where it participates in HOPS-catalyzed membrane fusion reactions. Syntaxin-17 has a unique double hairpin structure for insertion into the limiting membrane of the autophagosome, and mediates fusion with late endosomes and lysosomes in a VAMP8 and human synaptosomal-associated protein 29 (SNAP-29) regulated mechanism [172,173,174,175]. In flies, syntaxin-17 deficiency causes neurodegeneration due to the accumulation of autophagosomes [176]. Syntaxin-17 or SNAP29 deficiency in mammalian cells, and C-VPS/HOPS deficiency in drosophila, causes the accumulation of non-degradative autophagosomes, many of which still have a two-layered membrane, which suggests that syntaxin-17-SNAP29-VAMP8 interactions are primarily involved in fusion of autophagosomes with late endosomes [172,177]. Syntaxin17 also binds v-SNARE Vti1b and t-SNARE VAMP7 [172], and has been described to engage with endolysosomal VAMP7/SNAP29 in a HOPS-mediated fusion reaction of autophagocytosed mitochondria with endolysosomes [178]. The alternative SNARE Vti1b is involved in homotypic fusion of smaller autophagosome precursors [136], but also contributes at late stages of canonical autophagy and xenophagy (autophagy of pathogens) by pairing up with lysosomal VAMP7 to mediate fusion [173]. Vti1b may function at a later stage than syntaxin-17 as Vti1b deficiency causes accumulation of both autophagosomes and amphisomes (with a mild penetrance in knock-out mice) [179].

The nutrient sensor mTOR regulates autophagy induction, but also the SNARE machinery involved in autophagosome-lysosome fusion is directly sensitive to cellular nutrient status. Thus, SNAP-29 is *O*-glycosylated in fed cells, which results in a diminution of its ability to mediate lysosomal membrane fusion, a phenomenon which is reversed upon starvation and deglycosylation [180].

Curiously, syntaxin-17 is also present on the phagophore [120]. As it turns out, two other ATG gene products, namely Atg14 and Atg17, participate in both phagophore nucleation as well as in fusion events at the level of autophagosome entry into the endolysosomal system.

Thus, in mammalian cells Atg14, an essential subunit of the Beclin-PI3K complex at the point of nucleation and expansion [181,182], has a secondary role at the stage of fusion with late endosomes by binding and priming the bivalent syntaxin17-SNAP29 SNARE complex for interaction with endolysosomal VAMP8 [183]. In contrast, stabilization of the full *trans*-SNARE, syntaxin17-SNAP29-VAMP8 complex, seems to reside with the Ectopic P-Granules Autophagy Protein 5 Homolog (EPG5), which tethers Syntaxin-17/SNAP29 to VAMP8 present on lysosomes [184]. Dimerized Atg14 binds directly to the core domain of syntaxin-17 [183], whereas EPG5 and another recently identified fusion regulatory protein, human pleckstrin homology domain-containing family M member (PLEKHM), are recruited to autophagosomes by LC3 binding [184,185].

Lastly, Atg17, in complex with other Atg proteins, acts at the stage of autophagy induction in yeast, but is also necessary for down-stream recruitment of the vacuolar SNARE Vacuolar morphogenesis protein 7 (Vam7), which mediates fusion with the yeast vacuole [186]. It remains to be seen whether this finding translates into the mammalian system.

It is at present unclear whether syntaxin17, potentially Vti1b, Atg14, or Atg17 activity is regulated as they remain with the maturing autophagosome, or whether they undergo dynamic dissociation/association cycles. The latter may pertain to syntaxin17 [172]. As Atg17 binds with Vamp7 to inhibit its SNARE activity, it is possible in this case, that down-stream posttranslational modification liberates SNAREs for function at the stage of fusion with endolysosomes [186].

The HOPS complex is a SNARE-interacting complex mediating membrane tethering and fusion reactions of late endosomes and amphisomes with lysosomes. In yeast, all membrane fusions with the vacuole (lysosome) are controlled by the c-VPS/HOPS complex, which also occupies a dominant regulatory node in mammalian membrane traffic [187]. HOPS has been identified as a binding partner for syntaxin-17, and all subunits of HOPS are required for clearance of autophagosomes in drosophila [176,177]. Embedded in different subunits, the HOPS complex has both Rab7 guanine nucleotide exchange factor activity, as well as Rab7 effector function. Rab7 is an important recruiter of HOPS to the limiting membrane, and RILP, a Rab7 effector and guardian of late endosomal structure and function [161], also recruits HOPS to late endosomes independently of Rab7 [188].

A mutation (D251E) of the HOPS subunit VPS33A, responsible for v/t-SNARE ligation, causes locomotor defects and purkinje cell death in mice (buff mutant mice). The mutation seemingly increases the affinity of HOPS interactions with the trans-SNARE complex of syntaxin-17/SNAP29/VAMP8 in a way that hinders fusion of autophagosomes with endolysosomes [189]. This study found endosomal trafficking to lysosomes to be unperturbed, and similarly, others have found that VPS16 subunit deficiency specifically affects the autophagosomal but not the endocytic maturation pathway [168]. In contrast, other groups find a dual block in both maturation pathways following deficiency of any HOPS subunit in mammalian cells [187] as well as drosophila [177] in line with the dominant regulatory role of HOPS in yeast.

Two different protein factors co-ordinately regulate the progression of autophagosomes and endosomes through the maturation pathway by modulating Rab7 and HOPS function. Beclin-1, present in the ULK initiator complex bound to ATG14L, can engage in alternative binding reactions with either UV radiation resistance-associated gene protein (UVRAG) [159] or Run domain Beclin-1-interacting and cysteine-rich domain-containing protein (Rubicon) [181,182], which mediate a positive or negative influence on autophagosome (and endosome) maturation, respectively. UVRAG, which displaces ATG14L from the initiator PI3K complex, interacts with the HOPS complex to stimulate Rab7 GTPase activity [159] and PI3K activity [182], and promote autophagosome fusion with late endosomes/lysosomes. UVRAG can be sequestered from the PI3K complex by Rubicon, which therefore acts as a negative regulator of endosomal and autophagosomal flux [160,170,181].

The terminal maturation of autophagosomes and/or amphisomes also requires ESCRT subunits [190]. Both in drosophila and mammalian cells, deficiency of ESCRT subunits causes a dramatic accumulation of autophagosomes, correlating with cytotoxicity, due to reduced endosomal and lysosomal fusion [191,192,193]. The requirement for ESCRT and HOPS subunits likely reflects the need for a continuous and uninterrupted flux of late endosomes/MVBs for fusion availability with autophagosomes [177,193].

PLEKHM, which was identified as an interaction partner of LC3B in the yeast two-hybrid system [139], has been shown to interact with the HOPS complex [185]. PLEKHM associates with Lysosome-associated membrane protein (LAMP)1-positive endolysosomes in resting conditions, and mediates vesicle tethering and autolysosome fusion reactions in interplay with Rab7 and Syntaxin17-SNAP29. Deficiency of PLEKHM inhibits degradation of both endocytic and autophagosomal cargo [185].

Fusion of autophagosomes with lysosomes requires reorganization of the local actin network. This function is upheld by HDAC6, which controls the actin modulating protein cortactin through deacetylation [194]. We have recently described that tubulin polymerization promoting protein (TPPP/p25α), a protein ectopically expressed in dopaminergic neurons in PD, blocks amphisome fusion with lysosomes [29], most likely by its ability to inhibit the deacetylase activity of HDAC6 [195].

## 5. Amyloids Are Substrates of the Autophagosomal Machinery

Pathological protein aggregates in the cytosol, including α-synuclein, are removed and degraded by the ubiquitin-proteasome system and different forms of autophagy [196,197,198]. Several studies have shown that pathological forms of α-synuclein [117,142,196,197,199], huntingtin [142,200,201,202], and tau [203,204,205,206] are substrates of the chaperone-mediated and the macro-autophagosomal pathways. Even though APP/Aβ is membrane-bound/luminal, the cellular levels of these proteins is in part regulated by autophagy [207], although this contention has been challenged [208]. Recent studies in mouse models of AD with Atg7 deficiency support a role of autophagy in secretion of Aβ_1–42_ and deposition of amyloid plaques [209]. It is conceivable that Aβ_1–42_-containing endosomes and/or lysosomes are dysfunctional and damaged, and as such become sequestered into autophagosomes [210,211].

While α-synuclein is taken up by both macroautophagy and chaperone-mediated autophagy, modified or aggregated forms of α-synuclein have also been found to partially inhibit the very same pathways in different neurodegenerative disease models [117,196,197,199,212,213]. The mechanisms range from inhibition of autophagosome formation [208,212,214,215], inhibition of cargo loading in selective autophagy [202] or chaperone-mediated autophagy [117,199], and deranged maturation typically by failure to fuse with lysosomes [191,192,193].

## 6. Late Endosomes and Amphisomes in Secretion of Amyloids

Regardless of the mechanism of amyloid import, the ionic and enzymatic milieu in late compartments of the endosomal and autophagosomal pathway could promote generation of misfolded or otherwise modified amyloids. Prelysosomal and lysosomal organelles have a low pH and a redox environment, which differs from other organelles of the endosomal or autophagosomal pathways. It is very possible that the chemical milieu of end stage organelles may promote formation of proteotoxic species of contained amyloids by some form of conformational chemistry. Thus, mildly acidic conditions greatly increase the propensity of α-synuclein to form aggregates by increasing fragmentation and secondary nucleation events hardly detectable at neutral pH [12]. Different cholesterol- or tetraspanin-organized microdomains of the limiting membrane of the MVB or the exosomes themselves may promote aggregation and proteopathic templating. For example, glycosphingolipids GM1 and GM3 contained in exosomes support α-synuclein aggregation in the lumen of the MVB [216]. It seems possible that qualitative conformational, chemical, or proteolytic alterations of amyloid may increase their transmission, uptake, or propensity to catalyse seeded aggregation after release from cells.

### 6.1. Unconventional Secretion

While it has been known for long that PrP^sc^ and Aβ_1–42_ are released from nerve cells, it has only more recently been recognized that also cytosolic amyloids can be secreted by one or more unconventional mechanisms. Unconventional secretion refers to the cellular release of proteins, which do not traffic through the normal ER-Golgi biosynthetic pathway (they lack a signal peptide), but use other means to reach and traverse the cell surface. The number of proteins known to leave cells unconventionally, as well as the number of unconventional pathways available for the purpose, are increasing steadily [217]. Other mechanisms than exocytosis could conceivably be involved in the secretion of amyloid protein from nerve cells including budding of microvesicles from the plasma membrane, nanotubules, chaperone assisted secretion of amyloid [218,219], or escape through pores formed in the membrane by amyloids [23], but here we will focus on mechanisms involving exocytosis of vesicular intermediates.

In the case of α-synuclein, several groups have documented unconventional mechanisms of secretion [28,29,30,32,33]. The group of Lee [32,33] were the first to describe unconventional secretion of α-synuclein aggregates, and also later reported that secretion was upregulated by cellular stress and depended on the aggregation state of α-synuclein. These authors proposed a vesicular exocytosis-model of α-synuclein secretion.

If so, the nature of the secretory organelle remains uncertain. Different studies have suggested early [31] or late endosomes [30] but without addressing the obvious topological barrier for crossing of cytosolic amyloid into the lumen of the endosomal pathway. Entry does occur though, as α-synuclein can be localized to lysosomal and prelysosomal compartments in the brain [198].

We believe that late endosomes and in particular amphisomes constitute attractive candidates for the dissemination of amyloid by exocytosis. First of all, the ESCRT-mediated import of ubuiqitinated cargo into ILVs in the case of late endosomes, or the direct uptake of cytosolic cargo into autophagosomes by selective autophagy, mechanistically explains the presence of amyloid in the organelle lumen. This is not counting chaperone-mediated autophagy, where the lysosomal membrane protein LAMP2a directly imports α-synuclein into lysosomes [117,197,199], nor microautophagy where cargo is imported into late endosomes [116]. Second, both late endosomes and amphisomes are known to undergo physiological exocytosis, a process which could be enhanced under stressful or pathological conditions [220]. Finally, the endosomal and autophagosomal pathways are linear pathways. Although membrane and proteins can enter and leave the endosomal pathway in vesicular carriers at several destinations along the maturation pathway, the bulk of the endosome or autophagosome continues to the terminal fusion with lysosomes. Therefore, any dysfunctional organelle along the route will cause an accumulation of incoming vesicles, which is often quite toxic to cells [177,192], and relief of cytotoxic burden requires, as a last resort of clearance, exocytosis of the accumulating organelle [29]. Because amphisomes and late endosomes are the penultimate organelles before lysosomes, they are particularly prone to exocytosis in the context of the omnipresent lysosomal deficiency noted in neurodegenerative disease. In the following, we discuss the role of MVB/late endosome and amphisome exocytosis in the secretion of amyloid.

### 6.2. Exocytosis of Amyloid by MVBs and Late Endosomes

MVB and late endosome exocytosis in mammalian cells is controlled by a number of small GTPases, including Rab11 [221], Rab27A and B [222] and their effectors, as well as Rab35 [223]. Recently, Ras-related protein Ral (Ral)A/B has also been implicated. Ral overexpression effects a centrifugal MVB distribution close to the plasma membrane [224], which is perhaps similar in action to Rab11 [221]. The t-SNARE syntaxin-5 may work down-stream of Ral to mediate fusion [224], perhaps in combination with the prevalent endosomal SNARE VAMP7, but the SNARE machinery required for MVB and late endosome fusion with the cell surface remains ill defined.

#### 6.2.1. MVB and Late Endosome Secretion of Aβ

Both PrP and APP are transmembrane proteins, and pathological alterations (PrP conversion to PrP^sc^ and APP cleavage) cause the extracellular exposure of PrP^sc^ or the luminal release of Aβ_1–42_ through exposure of APP to β- and γ-secretases. APP is known to shuttle between the trans-Golgi network (TGN) and endosomes [225,226,227]. While β-site cleavage of APP is accepted to occur in endosomes or on the cell surface, the precise localization of γ-secretase cleavage (within the transmembrane domain of APP) is under debate and has been proposed to occur in the TGN, biosynthetic vesicles [227], or in endosomes [225,226,228]. Assuming an endosomal origin of γ-secretase activity, it is interesting to note that APP and products of β- and γ-secretase activity, including Aβ_1–42_, can be localized to MVBs and exosomes [101,102,229,230], and the release of Aβ_1–42_ is increased by agents that prevent fusion with lysosomes [102]. As Aβ_1–42_ is produced and released into the lumen of late endosomes, there is no requirement for autophagy to explain the luminal disposition of amyloid and its secretion by exocytosis of late endosomes. Nevertheless, AD is the neurodegenerative disease where accumulation of autophagosomes and amphisomes is most conspicuous [231,232], and recently, autophagy has been tied to degradation and/or secretion of Aβ_1–42_ in mouse models and cell culture studies [207,209,214]. This suggests that injured endosomes containing amyloid may be specifically engulfed by autophagosomes [210,211], following endosomal membrane permeabilization due to luminal oligomer or protofibril formation.

#### 6.2.2. MVB and Late Endosome Secretion of α-Synuclein and Other Cytosolic Amyloids

Most amyloidogenic proteins associated with neurodegenerative disease are cytosolic proteins, and any role of late endosomes in the extracellular release of these protein aggregates requires crossing of topological membrane barriers.

In this respect, it has attracted attention that SOD, tau, and α-synuclein in mainly monomeric form associate with exosomes [26,28,30,34,35,233,234]. Exosomes are attractive candidates for transmission of amyloid species, because of their potential to fuse with the plasma membrane (or limiting endosomal membrane) of the recipient target cell leading to the intra-cytosolic release of amyloid species contained within the exosome lumen. Based on high ionic strength extraction, protease sensitivity, or ultrastructural analysis, amyloid is in part enclosed in the lumen of these exosomes, implying that amyloid, on the cytosolic aspect of the limiting membrane of MVBs, can be internalized and enclosed by ILV budding. It should be stressed that very small amounts of total secreted amyloid are associated with exosomes (<1%–2%) the great majority of released material consisting of soluble amyloid in monomeric or aggregated forms [29,229,235,236]. Exosome association of α-synuclein may depend on the cellular model as some groups, including our own, find no or very little association with secreted exosomes [29,31,237]. PrP^sc^ also associates with exosomes, and it has been shown that exosome-associated PrP^sc^ is processed by unique N-terminal proteolysis [235,236]. Exosomal fractions are capable of transmitting prion disease to cells in culture or to mice following intracerebral injection [235,236], and transmission of α-synuclein oligomers is enhanced by exosomes in vitro [28].

Histopathological analysis of disease propagation in the brain of mice [87] or man [1,86] show that amyloid transmission takes place along interconnected nerve fiber tracts indicative of trans-synaptic transmission. Mechanistic data to support this contention is still entirely lacking, but it is worthwhile to note that exosomes in other systems show propensity for trans-synaptic relay of protein factors. MVBs and late endosomes are known to localize to both pre- and post-synaptic compartments [230,238,239,240,241], and convincing evidence implicates an exosomal shuttle in the trans-synaptic delivery of wnt and the wnt-binding protein Evi in the neuromuscular junction of drosophila [241,242]. Exosomal transmission may also explain the experimental trans-synaptic transfer of tetanus toxin-conjugated Green Fluorescent Protein (GFP) (which is sorted to the ILVs of MVBs) between connected neurons [243] or the transmission of retrogradely transported trophic factors in between neurons [238]. The concentration of MVBs in synaptic structures is responsive to exogenous stimulation [240], and at least one study has demonstrated that exocytosis of MVBs and exosomes is regulated by nerve cell activity following glutaminergic stimulation [244].

Despite this precedence, the entry of amyloid into ILVs is only rudimentarily described, perhaps with the exception of one case, which we will describe below in some depth.

## 7. Neuronal Precursor Cell-Expressed Developmentally Downregulated 4 (Nedd4)-Mediated Import of α-Synuclein into ILVs?

As ubiquitination is required for ESCRT cargo import, it is interesting that α-synuclein has been recognized as a substrate of the cytosolic E3 ubiquitin-protein ligase Nedd4, which attaches K-63 linked ubiquitin chains to the C-terminus of α-synuclein [245,246]. In yeast models of α-synucleinopathy Nedd4 constitutes an important node, whose manipulation modifies toxicity of α-synuclein through alteration of membrane trafficking [247,248], and Nedd4 over-expression has proven beneficial in small organism and mouse models of PD [249].

Nedd family interacting protein 1 (Nfdip1) is an endosomal transmembrane adaptor protein, which interacts with Nedd4 and expands the repertoire of substrates available for ubiquitination by Nedd4. Nfdip1 localizes Nedd4 to late endosomal membranes, where Nedd4 also interacts with Alix [250] and possibly TSG101 [251,252] both associated with the ESCRT-I complex. Nfdip1 is required for the Nedd4-mediated ubiquitination and degradation of α-synuclein [245] and is secreted in exosomes [253]. In mammalian cells Nedd4-mediated relief of protein misfolding cytopathology seemingly involves an enhanced degradation of α-synuclein in a process, which requires the ESCRT complex [245,249] whereas amelioration of toxicity occurs without any alterations in cellular α-synuclein accumulation in yeast [247]. More experiments are clearly needed to address how Nedd4 modulates α-synuclein metabolism.

**Figure 2:**
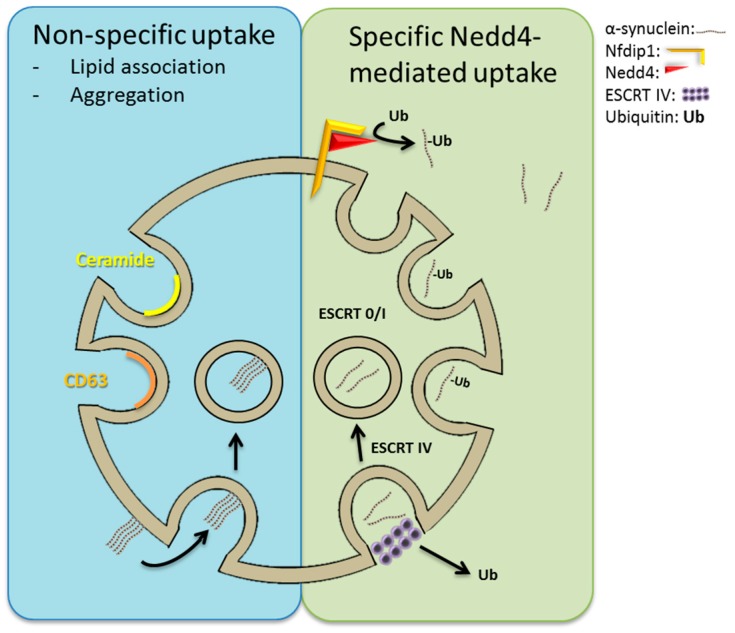
Import of cytosolic α-synuclein into intraluminal vesicles of multivesicular bodies and late endosomes. (**Right**) Nfdip1 recruits the E3 ubiquitin ligase Nedd4 to the limiting membrane of multivesicular bodies and late endosomes. Neuronal precursor cell-expressed developmentally downregulated 4 (Nedd4) ubiquitinates (Ub) α-synuclein, which thereby becomes a substrate of the Endosomal sorting complex required for transport (ESCRT) machinery for import into intraluminal vesicles (ILVs; exosomes upon secretion per convention). Secreted α-synuclein is not ubitiquitinated, as ubiquitin is removed by ESCRT-IV before scission of the budding intraluminal vesicle. Alternatively (**Left**), aggregated and lipophilic proteins, to which amyloids conform, can enter into ILVs by a mechanism that is independent of the ESCRT machinery. The circumstances of import, and whether this form of import involves alternative ceramide- or CD63-based forms of ILV formation, remain unknown.

Although Nedd4-mediated ubiquitination would drive the assimilation of α-synuclein into ILVs of late endosomes, ubiquitin is not necessarily needed to accomplish import. Intriguingly, it has been shown that the effect of higher-order oligomerization and membrane association is sufficient to confer entry into ILVs of diverse proteins [106,254], and it is possible that aggregated α-synuclein, which readily associates with membranes, may gain access to the lumen of ILVs by a similar mechanism (see Figure 2).

Although the total volume of cytosol enclosed by exosomes is considerable, it is likely not a mechanism for bulk disposal of cytosolic protein aggregates (size would be limiting), and is unlikely to account for the up to 10% of the total cellular α-synuclein pool that can be released from neurons [29]. In addition, it is clear in animal models of synucleinopathy and AD that exosomes are not required for the initiation of propagating inclusion body disease or amyloidosis, respectively, which can be inoculated by injection of purely synthetic amyloid into the brain [72,80]. In fact, in animal models of AD, the highest specific inoculating activity of proteopathy of fractionated brain tissue homogenates resides in the non-exosomal fractions [3]. Neither are exosomes required for the ability of nerve cells in culture to internalize recombinant α-synuclein [36,40,72] although some studies have shown a higher proficiency of exosome-mediated amyloid transfer than by fluid phase alone [28].

## 8. Exocytosis of Amyloid by Amphisomes

Autophagosomes and amphisomes appear as highly relevant compartments of amyloid secretion. Not only are cytosolic aggregates taken up directly by autophagy, thereby bypassing the topological barrier to vacuolar elements, but amphisomes can also undergo physiological exocytosis to release their contents [220,255], a process aggravated by lysosomal deficiency. Physiological unconventional secretion of protein factors by autophagy (dubbed autosecretion) was first demonstrated for Acetyl coenzyme A (Acyl-CoA) binding protein from yeast [255,256,257], and later it was shown that Interleukin-1 β (IL-1β) and High mobility box group 1 (HMBG1) are secreted by a similar mechanism in mammalian cells [220].

Autosecretion of IL-1β in human cells and Acyl-CoA binding protein in yeast depend on multiple Atg proteins and Golgi reassembly stacking protein (GRASP), a normally Golgi-localized protein, which also affects early steps in the autophagy pathway [128,220,255,256,257]. GRASP is a presumed membrane tethering factor, which not only participates in autosecretion but also in other forms of unconventional secretion including trafficking of certain membrane proteins to the cell surface [128,258]. In yeast the ESCRT-I subunit Vps23 (TSG101 homologue) and ESCRT-III subunit Vps4 are also required for autosecretion [255,257]. Whether this reflects that ESCRT components are directly involved in the autosecretory process or a requirement for an uninterrupted flow through the endosomal pathway for amphisome formation remains to be clarified. RalA/B, which promotes MVB/late endosome fusion with the plasma membrane [224], is also present on autophagosomes [259], but its involvement in exocytosis of amphisomes is unknown.

### Secretion of α-Synuclein by Exophagy

We have recently shown that α-synuclein in NGF-differentiated cathecholaminergic PC12 neurons is taken up by autophagy, and that this process is enhanced by aggregation of α-synuclein through co-expression of the PD-related protein TPPP/p25α [29,260]. TPPP/p25α also mimics aspects of lysosomal dysfunction by inhibiting final fusion of amphisomes with lysosomes by blocking HDAC6 activity [29]. This causes accumulation of amphisomes and an increased secretion of α-synuclein to the medium, which could be decreased by knocking-down Rab27A required for secretion of late endosomal elements [222]. We find that the bulk of α-synuclein monomer and high molecular weight aggregates are soluble and only a minute quantity of monomer is associated with exosomes [29].

In several different forms of unconventional secretion, a requirement for Rab8 has been noted [220,258]. The role of this small GTPase in unconventional secretion and autosecretion remains unsettled, but in other contexts Rab8 is involved in the vectorial vesicular trafficking to areas of polarized plasma membrane growth [261,262,263,264] and vesicle docking to acceptor membranes upstream of the SNARE machinery in fusion reactions [262]. Rab8 interacts with synaptotagmins also engaged by Rab27A [265]. Our finding that Rab8 colocalizes with mature autophagosomes and amphisomes, and greatly increases exophagosomal α-synuclein release concurrent with an amelioration of cytopathology and nerve cell survival [29], therefore offers a possible additional explanation to the suppressive effect of Rab8 on α-synuclein-induced toxicity in yeast, small organism models and mammalian cells in culture [266].

In yeast, plasma membrane-localized exocytic SNARE’s Sso1 and Sec9p (mammalian homologues Syntaxin-1 and SNAP25, respectively) are required for exocytosis of amphisomes [255,267]. However, the very same SNAREs are also required early in the autophagosomal pathway [268] making the effect of SNARE deficiency on autosecretion difficult to dissect from autophagy initiation. Further work is necessary to conclusively establish the sets of SNAREs that mediate exophagy in yeast and mammalian cells.

## 9. Lysosomal Deficiency

Lysosomal dysfunction and deficiency is a general cytopathic feature of neurodegenerative disease, and we have already discussed how this increases exocytosis of prelysosomal elements. In the following we will examine known types of lysosomal deficiency, exemplified mainly by identified genetic risk factors for the development of PD, and discuss lysosomal exocytosis. However, first we will high-light the intimate physiological relationship between lysosome dynamics and the autophagosomal pathway.

### 9.1. Lysosomes and Autophagy Are Closely Interrelated

Lysosomes and autophagy reciprocally affect each other [269], and many lysosomal and autophagosomal gene products are regulated coordinately by the master transcription factor EB (TFEB) [270,271]. TFEB is under normal conditions retained in the cytosol or bound to the limiting membrane of lysosomes, but following stress or starvation TFEB translocates to the nucleus where it promotes transcription of a network of genes required for autophagy and lysosomal function and regeneration [271]. Translocation requires that TFEB phosphorylation by active extracellular signal-regulated kinase (ERK) [271] or mTOR [272] is relieved in response to lysosome deficiency, low concentration of luminal amino acids (see below), or autophagy induction.

The mTOR complex localizes to the limiting membrane of lysosomes, and in an intricate mechanism, that has luminal amino acid sensing by the V-ATPase at its core, amino acid efflux from lysosomes in part governs its activation status [109,273,274]. mTOR activity is also regulated by the subcellular distribution of lysosomes, which are more peripheral in basal conditions but move towards the nucleus (and the microtubule-organizing center (MTOC)) in response to nutrient starvation correlating with an increased pH and mTOR activation [275]. It remains to be established how these results align with the recent demonstration that peripheral lysosomes are relatively devoid of Rab7 and V-ATPase, resulting in a higher pH [276]. In a distinct mechanism, mTOR reactivation at the termination of an autophagosomal response or following protracted starvation regulates the tubular reformation of (proto)lysosomes from autolysosomes [277].

### 9.2. Mechanisms of Lysosomal Deficiency in Neurodegenerative Disease

Dysfunction of the autophagolysosomal degradation pathway is a common feature of the genetic synucleinopathies [278], and there is little doubt that lysosomal deficiency is an important cytopathological mechanism in neurodegenerative disease [109,215,279,280,281,282].

**Figure 3:**
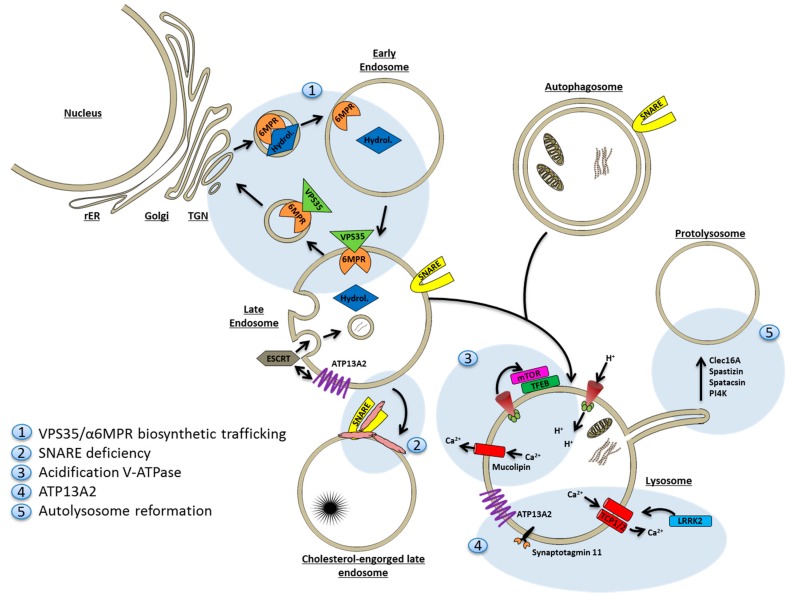
Mechanisms of lysosomal deficiency. (1) Normal delivery of the 50+ lysosomal acid hydrolases can become compromised by the lack of individual functional hydrolases or more generally by the derangement of Trans-Golgi network (TGN)-endosome trafficking affecting retromer (VPS35 mutations) function and its client Mannose-6-phosphate receptor (M6PR), the receptor for sorting of many hydrolases from TGN to the endosome; (2) In some lipid storage diseases SNAREs, required for entry of autophagosomes into the endolysosomal system, become “trapped” in cholesterol-enriched microdomains of lipid-laden late endosomes/lysosomes; (3) Proton and calcium fluxes are also affected in Alzheimer’s Disease (AD) and Parkinson’s Disease (PD). Presenilin (PSEN) mutations compromise sorting and/or function of the v-Adenosine Triphosphatase (ATPase) leading to relative alkalisation of the lysosome with reduced proteolysis. PSEN mutations also indirectly affect calcium efflux through mucolipin channels. The lysosomal phenotype contributed by Leucine-rich repeat kinase 2 (LRKK2) mutations can be rescued by Two-pore channel (TCP2) calcium channel knock-down; (4) ATP13A2 positively regulates ILV formation in late endosomes, and ATP13A2 mutations confer reduced luminal acidification and proteolysis that can be rescued by overexpression of synaptotagmin 11; (5) Autolysosome reformation is inhibited by gene products associated with neurological disease including clec16A, spastizin and spatacsin, and PI4K.

There are many conceivable ways lysosomes can fail to perform their normal duty as final degradation apparatus of endosomal and autophagosomal cargo (See Figure 3).

#### 9.2.1. Deficiency of Degradative Hydrolases

First of all, the delivery of mature degradative hydrolases to the lysosomal compartment may be compromised, or specific acid hydrolases may be absent all together. In the latter case different lysosomal storage disorders may arise. For example Gaucher’s disease caused by β-glucocerebrosidase deficiency, which incidentally constitutes the strongest genetic risk factor identified to date for development of PD. Even though genetic hydrolase deficiency affects only a single or few substrate(s), secondary lysosomal defects arise due to accumulation of substrate, and may extend to also involve autophagosomal pathways [237,283,284].

Several genes identified in large genome-wide association studies as being linked with development of PD are known or have proven to be involved in vesicular trafficking. Thus, VPS35 (PARK17) is a subunit of the retromer, an endosomal trimeric cargo-recognition protein complex composed of VPS26, VPS29 and VPS35, involved in recycling of receptors from late endosomes to the TGN. Retromer dysfunction was first described in AD [285], while continued studies are required to fully explain how VPS35 mutations result in α-synuclein accumulation, loss of dopaminergic nerve cells, and locomotor problems in mouse [286] or drosophila [286] models of PD. Client proteins of the retromer include M6PR [287], LAMP2a [286], and APP [225]. It is therefore not surprising that functional deficiency of VPS35 impairs lysosomal proteolysis, as acid hydrolase delivery would be expected to diminish due to sequestration and increased degradation of M6PR in endolysosomes [287]. M6PR is down regulated in brains from α-synuclein overexpressing animal models and in PD patients with early diagnosis [288]. Transport of hydrolases from the TGN to endosomes is directly affected by α-synuclein over-expression. Thus α-synuclein inhibits the activity of Rab1, a small GTPase necessary to promote biosynthetic vesicle transport from the TGN, in both yeast, small animal models, and human cells [266,289,290].

Also, autophagy is adversely affected by VPS35 deficiency. The relative lack of LAMP2a decreases chaperone-mediated autophagy [286], and macroautophagy is impeded by the failure to recruit the actin-organizing protein WAS protein family homolog 1 (WASH) to autophagosomes and endosomes via VPS35 binding [291]. Parkinsonian VPS35 mutations may both interfere with the sorting function of VPS35, as well as displace the retromer from late endosomes to cytosol [292,293].

#### 9.2.2. Sequestration of Fusion Machinery Components

SNAREs required for fusion of amphisomes or late endosomes with lysosomes may not be available. An example is several lysosomal lipid storage disorders, where autophagy is known to be impeded by a failure to enter the endolysosomal system due to the lack of SNAREs to catalyze the fusion reaction [294]. Instead, SNAREs are aberrantly sequestered in cholesterol-enriched domains on the limiting membrane of cholesterol-engorged late endosomes and lysosomes [295].

#### 9.2.3. Lysosomal Proton and Calcium Imbalances

The v-ATPase is responsible for proton pumping into the endolysosomal lumen to lower the pH to 4.5–5 required for optimal catalytic activity of the 50+ different lysosomal hydrolases.

In AD, where accumulation of autophagosome vacuoles is prominent [231], it has recently been proposed that Presenilin (PSEN), mutations of which cause familial AD, is required for proper acidification of lysosomes [215]. Mechanistically, PSEN was found to be required for proper *N*-glycosylation of a subunit in the v-ATPase, which mediates its sorting from TGN to lysosomes. Others have opposed this conclusion and find that PSEN mutations in drosophila cause calcium efflux from lysosomes through undescribed mechanisms [296]. Although calcium efflux (through non-selective cation mucolipin channels) is also true in mammalian cells, only correction of lysosomal pH, not calcium concentration, ameliorates lysosomal proteolysis and block in the autophagosomal pathway [297].

Lysosomal calcium homeostasis is also affected in PD. Leucine-rich repeat kinase 2 (LRRK2; PARK8) is a large protein with both kinase and GTPase activity. It suppresses autophagy in normal conditions [298,299], but mutations have been tied to alterations in late endosomal membrane trafficking [300] and the formation of pathologically large lysosomes [301,302]. Vps35 overexpression rescues the pathological phenotype of LRRK2 mutations in drosophila models [303]. The lysosomal phenotype is specifically mediated through cation two-pore channels (TPCs) that gate calcium in the lysosome; silencing of TPC2 corrects the LRRK2 phenotype [301].

#### 9.2.4. ATP13A2

ATP13A2 (PARK9) is a lysosomal type 5 P-type ATPase that takes part in the control of lysosomal acidification. ATP13A2 has been linked to autosomal recessive early-onset parkinsonism, pyramidal degeneration and dementia. Cells carrying deficient ATP13A2 have impaired lysosomal acidification, reduced degradation of lysosomal substrates, and diminished clearance of autophagosomes [304,305]. ATP13A2 localizes to lysosomes and MVBs where its activity is supported by binding inositol lipids in the limiting membrane [306]. ATP13A2 has been shown to positively regulate exosome biogenesis through interactions with the ESCRT machinery, and overexpression of ATP13A2 increases the content of α-synuclein secreted in exosomes several-fold [233,234]. The common LRRK2 G2019S mutation causes formation of enlarged lysosomes with a reduced luminal pH, and an increased complement of ATP13A2 in the membrane [302]. ATP13A2 depletion negatively regulates synaptotagmin 11 (SYT11), another putative PD-associated gene, at both transcriptional (through TFEB regulation) and post-translational levels [307]. Overexpression of SYT11 rescues the ATP13A2 lysosomal phenotype, and conversely, SYT11 knock-down is sufficient to induce lysosomal dysfunction and impaired degradation of autophagosomes.

#### 9.2.5. Faulty Lysosome Regeneration

Another mode of lysosomal deficiency arises when autolysosomes fail to regenerate at the end of the autophagosomal response. Normally, protolysosomes are regenerated from autolysosomes in an mTOR-dependent mechanism [277]. At this point mTOR reactivation is required, and it has been described that spinster, a putative carbohydrate permease in lysosomes, is required for reactivation of mTOR and initiation of lysosome reformation following starvation [308]. Slender tubules shaped by kinesin motor force [309] are formed and then pinched off the limiting membrane to form protolysosomes in a process requiring clathrin and phosphatidylinositol-4,5-bisphosphate [310]. Phosphatidylinositol-4-phosphate (PI4P) can serve as precursor for the latter and is generated by PI4IIIK. However, PI4IIIK specifically seems to affect the membrane fission step of tubule formation suggesting a role for PI4P in this process [311].

Recently, Clec16A, a protein associated with development of multiple sclerosis, has been shown to be required for autolysosome function in mouse models: in the absence of Clec16A both neuronal endocytosis and autophagy initiation are unperturbed, however, autolysosomes accumulate due to lacking regeneration of protolysosomes [312]. In particular, Purkinje nerve cells were affected.

Along the same lines, the two most common autosomal recessive hereditary spastic paraplegia gene products, the SPG15 protein spastizin and the SPG11 protein spatacsin, are both required for autophagic lysosome reformation [313,314]. Knock-out of these genes in mice cause accumulation of autolysosomes correlating with depletion of functional lysosomes, and leading to the development of a paraplegia-like phenotype with degeneration of corticospinal axons and loss of cortical neurons and, again, Purkinje cells.

### 9.3. Exocytosis of Amyloid by Lysosomes

It was once thought that lysosome exocytosis was the prerogative of certain specialized cell types containing so called lysosome related organelles. However, it is now recognized that all cells have the capacity for lysosome exocytosis in a calcium-dependent manner [315,316], and that lysosomal exocytosis can function as an integral process in excretion of undesirable and toxic materials [317]. The lysosomal fusion reaction with the plasma membrane depends on calcium sensor synaptotagmin VII [318] and VAMP7 on lysosomes, which binds syntaxin4 and SNAP23 in the plasma membrane [319]. It has been shown that TFEB (transcriptionally) promotes lysosomal exocytosis by increasing the population of plasma membrane-proximal lysosomes, and by increasing lysosomal calcium efflux through mucolipin channels [320]. Overexpression of TFEB caused an amelioration of lysosomal storage disease presumably by clearance of accumulated material through exocytosis. However, the conclusion is difficult to draw because TFEB also boosts the autophagolysosomal system in general. The recent demonstration in mouse models of AD that congregation of axonal lysosomes with a greatly reduced content of acid hydrolases surround amyloid plaques at all stages of formation [321] sustains the idea that lysosome exocytosis also in neurodegeneration is a mechanism of excretion of amyloid.

## 10. Microglia in Disease Dissemination

There is a huge body of in vitro and in vivo evidence demonstrating that neurodegenerative diseases are not cell-autonomous, but depend on the recruitment and activation of glia cells, first and foremost microglia, which are essential for progression of brain disease in animal models of PD and other brain diseases [322]. There is microglia activation before manifest nerve cell loss in man, primate, and mouse models, and amyloids in oligomeric or insoluble format are known to activate resident microglia through a variety of pattern recognition receptors including RAGE, CD36, TLRs, and FcγR’s [52,53,57,58,323,324,325], which drive classical activation of microglia (inflammation and pathogen combat). The ensuing chronic inflammation of the brain and/or cessation of neurotrophic support instill a milieu, which aggravates nerve cell death and promotes disease progression while impairing neuroregeneration [322,326].

Recently, potential new facets of microglia contribution to disease progression have been proposed. Thus, we have shown in vitro that microglia activated by conditioned medium from synucleinopathic neurons in culture (or LPS) increase α-synuclein secretion from the neurons [327]. While the microglia factors that mediate this effect are unknown, the ensuing neuronal hyper-secretion of α-synuclein depends on c-Jun N-terminal kinase (JNK) activation in the nerve cells [327]. Additionally, microglia might potentially ‘qualitatively enrich’ amyloids for their ability to transmit misfolding disease. Thus, it has recently been shown that microglia internalize tau by endocytosis, and then, subsequent to immune cell activation (by LPS), release tau contained within the lumen of exosomes [328]. In a mouse model of tauopathy propagation from entorhinal cortex to the dentate gyrus both microglia depletion and inhibition of exosome formation inhibits spread of cytopathology. What the study does not address is how internalized, soluble tau escapes from endosome to cytosol, is seemingly ubiquitinated, and then taken up by ceramide-based (and therefore ubiquitin-independent) ILVs before release by late endosome exocytosis. More work is clearly needed to understand how microglia can forward brain disease by promoting seed dissemination.

## 11. Aging, Proteostasis, and Autophagy

It is clear that aging constitutes a major predisposing factor for the development of protein misfolding disease. Remarkably, in *Caenorhabditis elegans* (*C. elegans*), several hundred proteins undergo a transition to insoluble form in aged worms [329,330]. Like human amyloids these proteins adopt a parallel β-pleated sheet conformation in their aggregated form, and a relatively high proportion of them are involved in energy production and translation, and have an effect on longevity. It is not known whether protein aggregation is a cause or an effect of normal aging, but interestingly, in the nematode models insolubility and aggregation could be circumvented by reduced insulin/IGF-1-signaling [330] known to extend life span. Reducing the propensity for protein aggregation chemically by treating *C. elegans* with thioflavin, which binds many amyloids, extends lifespan and slows ageing in processes, which depend on molecular chaperones, autophagy, and proteasomes [331].

Conversely, there is a large body of evidence to suggest that upregulation of the cellular degradative machinery, not only prevents protein aggregation, but also prevents neurodegenerative disease, and in some cases extends life span. Dietary restriction extends life span of organisms from worms to mammals by reducing insulin/IGF-1-signaling, mTOR activity, and mitochondrial respiration. In this respect, it is interesting to note that suppression of crucial autophagy genes in *C. elegans* cancels the effect of all known paradigms of life span extension [332]. mTOR also regulates life span of mammalian organisms. In mouse models rapamycin, an inhibitor of mTOR, and thereby activator of autophagy, extends life span of mice of both genders, even when treated with the drug quite late in life [333].

In general, protein homeostasis is perturbed across different tissues of aging humans [334]. A diminished capacity of the major degradative systems, the proteasome and the autophagolysosomal pathway [335,336], shifts the balance between production and clearance of endogenous noxious materials. The resulting accumulation of proteotoxic waste eventually ramifies to affect biosynthesis with development of ER stress and induction of the unfolded protein response (UPR) [337]. Not many studies have examined the workings of the autophagosomal system in the aging human brain. However, there is consensus that autophagosomal flux is reduced compared to young individuals as transcription of key players of the autophagosomal pathway including beclin-1, ATG5, and ATG7 is reduced [338]. AD stands out as the brain disease with the most prominent concentration of autophagosomal vacuoles [231]. In accordance herewith, many autophagy-related proteins participating on all levels of the autophagosomal pathway are upregulated in AD brain compared to age-matched controls [232,338]. An exception is beclin-1, which is down-regulated in AD brain [214,232,339]. In PD there is no information on the macroautophagosomal system, but chaperone-mediated autophagy is depressed by the down-regulation of LAMP2a and Hsc70 [340].

## 12. Therapeutic Implications

The transmission model of neurodegenerative disease propagation opens up for entirely new ways of thinking about therapeutic intervention. Evidence from animal model experiments indicates that upregulation of autophagy is beneficial in different types of neurodegenerative disease. In mouse models of PD, overexpression of beclin-1 ameliorates disease by correcting autophagosomal aberrations induced by α-synuclein expression [339]. In AD models, where beclin-1 expression is reduced, lentiviral overexpression of beclin-1 has also been reported to rescue cytopathology and reduce both intracellular and extracellular amyloid pathology in APP transgenic mice. Conversely, mice heterozygous for beclin-1 shows accelerated neurodegeneration [214].

The therapeutical promotion of autophagy alone is, however, most likely not a universal mechanism for correction of cellular pathology by misfolding. In the 5xFAD mouse model of AD, lysosomal dysfunction (acidification), Aβ deposits, and cognitive deficits were reduced by a GSK-3 inhibitor under conditions of mTOR reactivation and thus decreased autophagy [279]. Similarly, although perhaps coincidental, several drugs in clinical trial for treatment of AD actually reduce autophagosomal flux [338]. Finally, tissue specific and conditional knock-out of ATG7 in mouse models of AD decreases extracellular Aβ plaque burden by decreasing exocytosis of amyloid [209].

In particular, AD stands out as a neurodegenerative disease where autophagy enhancement could turn out to be problematic despite positive results gained with beclin overexpression in one mouse model of AD [214]. There is a forming consensus that the major cytopathic problem in AD is lysosomal clearance of autophagosomal cargo, rather than the workings of the autophagosomal pathway itself. By comparison of nondemented controls with early or late stage AD, evidence has been provided that the autophagosomal pathway is induced in CA1 pyramidal neurons of hippocampus in AD cases [232]. Although lysosomal biogenesis is also upregulated, the lysosomal capacity is exceeded by the inflow of autophagosomes causing the pronounced accumulation of autolysosomal vacuoles characteristic of AD [231,232].

Therefore, induction of autophagy is only relevant in disease states that do not involve lysosomal deficiency, as accumulation of prelysosomal elements would be exacerbated drastically and thereby toxicity increased.

Modulation of TFEB, the major transcriptional regulator of lysosomal and autophagosomal genes, to coordinately upregulate both autophagy and lysosomal biogenesis and recycling pose an appealing possibility. Since TFEB coordinately regulates both the autophagosomal pathway as well as lysosomal biogenesis there is a good chance that the plasticity of the network will be able to accommodate differences in cellular pathology in between different amyloids and on different patient backgrounds. TFEB has shown efficacy in suppressing amyloid toxicity in *C. elegans* as well as murine models of PD [341,342], and of AD and tauopathy [343]. Incidentally, TFEB overexpression increases longevity of *C. elegans* [341], again high-lighting the presumed, but not proven, causal correlation between autophagy, protein homeostasis, and life span. However, these data derived from animal models of neurodegenerative disease may not allow sufficient time for potential adverse effects of TFEB to be registered. A major concern in the human situation remains the propensity of TFEB to increase lysosomal exocytosis [320], which would support the amyloid dissemination step of transmission.

## 13. Conclusions and Perspectives

Lysosomal dysfunction, whether a primary or secondary deficiency, is an integral cytopathological trait of neurodegenerative diseases. While the mechanisms that precipitate lysosomal dysfunction may vary, the consequence is the same: a reduced proteolysis of undesirable cargo, whether caused by deficiency of acid hydrolase activity, or by a failure to fuse with incoming prelysosomal elements.

The resulting accumulation of amphisomes and/or late endosomes will eventually result in either cell death, or, as a last resort, the exocytosis of the accumulating organelle. Exocytosis of amphisomes relieves α-synucleinopathy in cultured cells, and increasing exocytosis, for example by expression of Rab8, further decreases synucleinopathic toxicity [29]. In storage disorder diseases, exocytosis of lysosomal elements ameliorates disease [320].

We have devoted a relatively large body of text to describe the SNAREs and regulatory factors that govern either the fusion of amphisomes/late endosomes with lysosomes, or the alternative fate, fusion with the plasma membrane to release contents to the surroundings. There is little doubt that this bifurcation in membrane trafficking pathways will be an important node for therapeutic modulation of amyloid clearance. The interesting results achieved by the group of Lindquist [247] lends credence to the belief that drugs that modulate this bifurcation can be identified: the unbiased library screen identification of small chemicals that affect the membrane trafficking node controlled by Nedd4, ameliorates synucleinopathy in yeast and mammalian neurons.

Very likely, nerve cells are able to concomitantly secrete both monomeric, but potentially modified, amyloid enclosed in exosomes, and soluble amyloid in the form of proteotoxic oligomers and aggregates [21]. In the future, it will be important to determine which of these species are most relevant for inoculation and disease propagation to devise focused therapeutic strategies.

## Abbreviations

Aβ	Amyloid β
Acyl-CoA	Acetyl coenzyme A
AD	Alzheimer’s Disease
AMPK	AMP-activated protein kinase
APP	Amyloid precursor protein
ARF6	Adenosine diphosphate-ribosylation factor 6
ATG	Autophagy-related
ATPase	Adenosine Triphosphatase
BAG	Bcl-2 associated athanogene
*C. elegans*	*Caenorhabditis elegans*
ER	Endoplasmic reticulum
EPG5	Ectopic P-Granules Autophagy Protein 5 Homolog
ESCRT	Endosomal sorting complex required for transport
FcγR	Fc receptors for IgG
GABARAP	GABA(A) receptor-associated protein
GFP	Green fluorescent protein
GRASP	Golgi reassembly stacking protein
GTPase	Guanosine triphosphatase
HDAC6	histone deacetylase 6
HMBG1	High mobility group box 1
HOPS	Homotypic fusion and protein sorting
Hrs	Hepatocyte growth factor-regulated substrate
ILV	Intraluminal vesicle
IL-1β	Interleukin-1 β
LAMP	Lysosome-associated membrane protein-2
LC3	Microtubule-associated proteins 1A/1B light chain 3B
LRRK2	Leucine-rich repeat kinase 2
M6PR	Mannose-6-phosphate receptor
MTOC	Microtubule-organizing center
mTOR	Mechanistic target of rapamycin
MVB	Multivesicular body
NBR1	Neighbor of BRCA1 gene 1
NDP52	Nuclear domain 10 protein 52
Nedd4	Neuronal precursor cell-expressed developmentally downregulated 4
Nfdip1	Nedd family interacting protein 1
PD	Parkinson’s Disease
PI(3/4)P	phosphatidylinositol (3/4)-phosphate
PI3K	Phosphoinositide 3-kinase
PI4K	Phosphatidylinositol 4-kinase
PLEKHM	Pleckstrin homology domain-containing family M
PSEN	Presenilin
PrP(^sc^)	Prion protein (scrapie associated)
RAGE	Receptor for advanced glycation endproducts
Ral	Ras-related protein Ral
RILP	Rab interacting lysosomal protein
RUBICON	Run domain Beclin-1-interacting and cysteine-rich domain-containing protein
SNAP	Synaptosomal-associated protein
SNARE	Soluble *N*-ethylmaleimide-sensitive factor-attachment protein receptors
SOD	Superoxide dismutase
STAM	Signal-transducing adaptor molecule
SYT11	Synaptotagmin 11
TFEB	Transcription factor EB
TGN	Trans-Golgi network
TLRs	Toll-like receptors
TPC	Two-pore channel
TPPP	Tubulin polymerization-promoting protein
ULK	The ser/thr-kinase uncoordinated family member-51-like kinase
UVRAG	UV radiation resistance-associated gene protein
Vam7	Vacuolar morphogenesis protein 7
VAMP	Vesicle-associated membrane protein
Vti1b	Vesicle transport through interaction with t-SNAREs homolog 1B
(C-)VPS	(Class C-) Vacuolar protein sorting-associated protein
